# Nonlinear relationship between circulating homocysteine and parathyroid hormone among patients with osteoporotic fractures: A cross-sectional study based on a hospital cohort

**DOI:** 10.1371/journal.pone.0349418

**Published:** 2026-06-01

**Authors:** Jia-hao Wang, Wei-hong Wang, Da-wei He, Ke Lu, Chong Li, Yan-ming Hao

**Affiliations:** 1 Department of Orthopedics, Gusu School, Nanjing Medical University–The First People’s Hospital of Kunshan, Suzhou, Jiangsu, China; 2 Department of Orthopedics, Kunshan Hospital Affiliated to Jiangsu University, Suzhou, Jiangsu, China; 3 Kunshan Women and Children’s Healthcare Hospital, a branch of Kunshan First People’s Hospital Group, Suzhou, Jiangsu, China; 4 Kunshan Biomedical Big Data Application and Innovation Laboratory, Suzhou, Jiangsu, China; 5 Department of Orthopedics, Traditional Chinese Medicine Hospital of Kunshan, Suzhou, Jiangsu, China; University of Life Sciences in Lublin, POLAND

## Abstract

Osteoporotic fractures are frequent among older adults and are often accompanied by disturbances in endocrine and metabolic regulation. Parathyroid hormone (PTH) serves as a key regulator of bone remodeling, while elevated plasma homocysteine—an established marker of vascular injury and skeletal fragility—has been implicated in osteoporosis. Yet, the interaction between these two biochemical factors remains insufficiently characterized. This study investigated the link between circulating homocysteine and serum PTH concentrations among 2,190 hospitalized patients with osteoporotic fractures, emphasizing nonlinear associations and modifying influences. Fasting blood samples obtained at admission were used to measure plasma homocysteine and intact PTH levels. Multiple regression and spline-based modeling techniques were employed to estimate independent relationships and potential threshold effects, with subgroup analyses considering hypertension, renal function, and magnesium status. Homocysteine demonstrated an independent, positive correlation with PTH (P < 0.001). A nonlinear dose–response relationship was observed, wherein PTH increased progressively with homocysteine up to about 18 μmol/L, followed by a sharper rise beyond this point, suggesting a statistically significant threshold. The relationship was more evident among patients with hypertension, impaired renal function, or low magnesium levels, indicating that vascular strain, renal stress, and mineral imbalance may heighten PTH activity. Elevated homocysteine levels may be associated with higher PTH concentrations in individuals with osteoporosis. However, the clinical relevance of homocysteine in metabolic assessment and risk stratification requires further validation.

## Introduction

Osteoporotic fragility fractures represent a major cause of disability and mortality worldwide, occurring roughly once every few seconds and affecting over eight million individuals each year [1]. Epidemiological estimates indicate that nearly one-third of women and one-fifth of men aged over 50 years will experience such fractures during their lifetime, emphasizing osteoporosis as a pressing global health issue [2]. Recent research highlights that skeletal fragility is not only a localized bone disorder but also intertwined with systemic metabolic and vascular dysregulation, underscoring the importance of discovering biomarkers that connect endocrine and bone metabolism [3].

Parathyroid hormone (PTH) is central to calcium regulation and bone remodeling processes [4]. Persistently elevated PTH levels—whether due to primary hyperparathyroidism or secondary causes such as vitamin D deficiency or renal impairment—promote excessive bone turnover and contribute to skeletal loss [5]. Moreover, elevated PTH has been associated with increased cardiovascular and vascular stiffness risk, implying endocrine influences beyond bone metabolism [6].

Homocysteine, a sulfur-containing amino acid recognized for its role in cardiovascular pathology, has also been implicated in skeletal fragility [7]. Epidemiological and mechanistic studies suggest that high homocysteine concentrations correlate with reduced bone mineral density and elevated fracture risk, likely through impaired collagen cross-linking, enhanced osteoclast activation, and diminished bone matrix integrity [8–9]. However, despite shared metabolic and vascular pathways, the interplay between homocysteine and endocrine regulators such as PTH remains poorly defined [10]. Preliminary data further suggest that renal and vascular function may affect both biomarkers, yet the nature of this relationship—linear or threshold-dependent—has not been fully clarified [11–13].

In this context, the present study explored the relationship between circulating homocysteine and serum PTH in a large cohort of 2,190 osteoporotic fracture patients, focusing on potential nonlinear associations and clinical modifiers. We hypothesized that increased homocysteine levels would be independently linked with higher PTH concentrations, particularly in the presence of vascular or renal stress. Understanding this relationship may help identify patients predisposed to secondary hyperparathyroidism and provide additional insight into metabolic disturbances in osteoporosis care [14].

## Materials and methods

### Research participants and design

This retrospective cross-sectional analysis utilized the electronic medical database of Kunshan Hospital, a tertiary medical center located in Jiangsu Province, China. Patients diagnosed with osteoporotic fractures (OPFs) between January 2017 and March 2024 were identified and reviewed.

Inclusion criteria were: (1) age ≥ 50 years; (2) diagnosis of primary osteoporosis based on clinical judgment or bone densitometry; and (3) documentation of a low-energy fragility fracture.

Eligible fracture locations included the hip (femoral neck, intertrochanteric, or subtrochanteric), thoracic or lumbar vertebrae, proximal humerus, and distal radius (wrist)—representing the most frequent patterns of osteoporotic fractures [15].

Osteoporosis was confirmed if: (1) a fragility fracture occurred with a bone mineral density (BMD) T-score ≤ –2.5 at the lumbar spine or hip; or (2) the BMD T-score was ≤ –2.5 without secondary causes of bone loss, even in the absence of a recorded fracture [16].

Exclusion criteria were as follows: (1) high-energy or pathological fractures; (2) severe comorbidities, such as primary hyperparathyroidism, prior parathyroid or thyroid surgery, end-stage renal failure requiring dialysis, active malignancy under chemotherapy, or advanced hepatic dysfunction; (3) missing values for major variables (homocysteine or PTH); (4) incomplete biochemical data (e.g., serum calcium, albumin, or creatinine); (5) missing demographic information (sex, age, or BMI); and (6) unavailable lifestyle variables (smoking or alcohol consumption).

**Fig 1:**
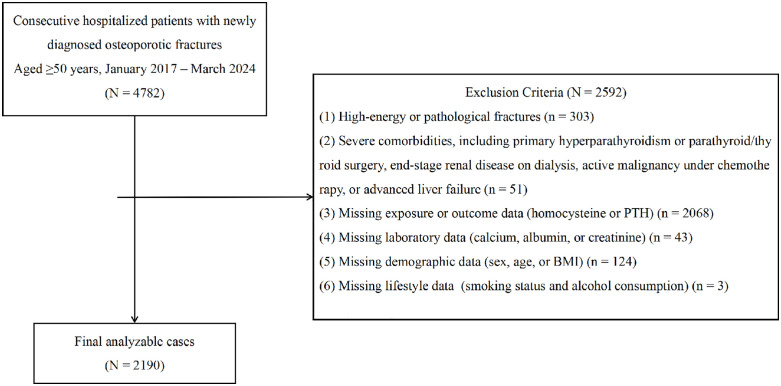
Flowchart of inclusion and exclusion for osteoporotic fracture patients.

Out of 4,782 initially screened fracture cases, 2,592 were excluded according to these criteria, leaving 2,190 patients eligible for final analysis. The age of the included participants ranged from 50 to 97 years. The study population flow is summarized in Fig 1.

This retrospective study was approved by the Ethics Committee of the Affiliated Kunshan Hospital of Jiangsu University (Approval No. 2024-03-053-H00-K01). The clinical data were accessed for research purposes between 30 November 2024 and 30 December 2024. Identifiable patient information was accessed only during the initial data extraction phase within the hospital information system. All subsequent data processing and statistical analyses were conducted using fully anonymized datasets. The authors did not have access to any information that could identify individual participants during or after data analysis.

### Exposure and outcome variables

Preoperative fasting venous blood samples were obtained within 48 hours of hospital admission to minimize perioperative metabolic variability. Serum homocysteine (Hcy, μmol/L) concentrations were determined by an enzymatic cycling method using a Cobas 8000 modular analyzer (Roche Diagnostics, Mannheim, Germany) [17].

Because Hcy values exhibited a right-skewed distribution, they were log-transformed (lnHcy) for analysis and treated as the main exposure variable.

The outcome variable was serum intact parathyroid hormone (PTH, pmol/L), measured by electrochemiluminescence immunoassay (ECLIA) on the same analyzer, with a reference interval of 1.6–6.9 pmol/L [18]. PTH values were right-skewed; therefore, the distribution was evaluated and a sensitivity analysis was conducted using ln-transformed PTH. The associations observed with lnPTH were consistent with the primary analyses, while the threshold effect was assessed using the original PTH values to preserve clinical interpretability.

All biochemical assays were performed by trained technicians following standard internal and external quality-control procedures.

### Covariates

Demographic covariates included age, sex, and body mass index (BMI), which was computed as weight (kg) divided by height squared (m²).

Clinical factors comprised hypertension, diabetes mellitus, smoking habits, alcohol intake, and the American Society of Anesthesiologists (ASA) physical status classification.

Laboratory indicators encompassed serum creatinine (Cr), albumin, and magnesium levels. The season of hospital admission was also recorded to capture possible seasonal variation in vitamin D levels and bone metabolism.

Smoking was defined as current or recent smoking within the past 12 months, while alcohol intake referred to drinking on at least one occasion per week during the preceding year.

All biochemical data were obtained from fasting samples collected within 48 hours of admission, and analyzed by trained laboratory staff under standardized quality-control protocols.

Covariates were selected a priori according to previous research that supports their biological relevance to both homocysteine metabolism and parathyroid hormone regulation.

### Statistical analyses

Continuous variables that followed an approximately normal distribution were summarized as means ± standard deviations (SDs), whereas non-normally distributed variables were reported as medians with interquartile ranges [Q1, Q3]. Categorical variables were presented as counts and percentages. For group comparisons, the Student’s t test was applied to normally distributed variables, the Mann–Whitney U test to skewed data, and the Pearson chi-square or Fisher’s exact test to categorical data, as appropriate. To assess baseline differences across exposure levels, participants were categorized into tertiles of natural log-transformed homocysteine (lnHcy).

One-way ANOVA and Kruskal–Wallis tests were used to examine trends for variables with normal and non-normal distributions, respectively. Initially, univariable linear regression was performed to explore the association between serum PTH (dependent variable) and each potential covariate (age, sex, BMI, smoking, alcohol intake, hypertension, diabetes, albumin, creatinine, magnesium, and admission season). Covariates with P ≤ 0.10 or those that changed the lnHcy–PTH coefficient by ≥10% were subsequently entered into multivariable regression models. Variance inflation factors (VIFs < 5) were considered indicative of acceptable collinearity.

The independent association between lnHcy and PTH was estimated using generalized estimating equations (GEE) with an identity link function and robust standard errors. Three hierarchical models were constructed: Model 1: unadjusted; Model 2: adjusted for age, sex, and BMI; Model 3: fully adjusted for smoking, alcohol intake, hypertension, diabetes, albumin, creatinine, magnesium, American Society of Anesthesiologists (ASA) class, and admission season. Regression results were expressed as β coefficients with 95% confidence intervals (CIs), where a positive β indicated a rise in PTH with increasing lnHcy levels.

To evaluate nonlinear patterns, generalized additive models (GAMs) with smoothing splines were fitted. When the smoothed curve suggested a nonlinear relationship, a two-piecewise linear regression (threshold-effect model) was conducted to determine the inflection point (knot). Linear relationships on both sides of the knot were estimated separately, and log-likelihood ratio tests were used to compare the two-segment model with the single-line model. The stability of the inflection point was verified using 1,000 bootstrap resamples.

Subgroup analyses were carried out to examine the robustness of the lnHcy–PTH association across predefined strata, selected based on biological relevance and prior literature. Subgroups included demographics (age, sex, BMI), lifestyle factors (smoking and alcohol), comorbidities (hypertension, diabetes, ASA class), biochemical indices (albumin, magnesium, creatinine), and admission season. Age was classified as ≤70 or >70 years; BMI as <25 or ≥25 kg/m²; ASA class as 0–1 or ≥2; and admission season as spring, summer, autumn, or winter. Laboratory indices were divided according to clinical cutoffs (e.g., albumin or magnesium: low vs. normal; creatinine: < 80 vs. ≥ 80 μmol/L). Interaction terms were incorporated into GEE models, and P for interaction < 0.05 was considered statistically significant.

All analyses were conducted using R software (version 4.0.5; R Foundation for Statistical Computing, Vienna, Austria) and EmpowerStats (X&Y Solutions, Boston, MA, USA).

Given the exploratory purpose of subgroup analyses, interaction results were interpreted cautiously rather than confirmatively.

## Results

### Baseline profiles of the study population

A total of 2,190 patients with osteoporotic fractures were included (mean age: 68.97 ± 10.97 years; 75.2% female). Participants were categorized into tertiles according to ln-transformed homocysteine levels (T1–T3). As shown in Table 1, serum creatinine and uric acid increased progressively with higher Hcy tertiles (both P < 0.001), while phosphorus slightly declined (P = 0.05). Calcium and magnesium showed no significant differences (P > 0.05). PTH concentrations rose markedly from 7.95 ± 2.02 pmol/L in T1 to 21.07 ± 11.21 pmol/L in T3 (P < 0.001). Other clinical factors, including age, BMI, hypertension, diabetes, smoking, and alcohol use, were comparable across groups. These results suggest that elevated Hcy levels were accompanied by higher creatinine levels and higher PTH concentrations among patients with osteoporotic fractures.

**Table 1:** Baseline demographic and clinical features of 2,190 individuals with osteoporotic fractures stratified by ln-transformed homocysteine levels.

Characteristics	Total	T1 (n = 730)	T2 (n = 730)	T3 (n = 730)	*P* value
Age, years	68.97 ± 10.97	68.99 ± 11.12	68.65 ± 10.81	69.25 ± 10.98	0.66
Sex, *n* (%)					0.83
Female	1647 (75.21%)	544 (74.52%)	549 (75.21%)	554 (75.89%)	
Male	543 (24.79%)	186 (25.48%)	181 (24.79%)	176 (24.11%)	
BMI, kg/m²	23.26 ± 3.28	23.26 ± 3.43	23.35 ± 3.27	23.16 ± 3.14	0.54
Hypertension, *n* (%)					0.66
No	1889 (86.26%)	629 (86.16%)	636 (87.12%)	624 (85.48%)	
Yes	301 (13.74%)	101 (13.84%)	94 (12.88%)	106 (14.52%)	
Diabetes, *n* (%)					0.97
No	2088 (95.34%)	696 (95.34%)	697 (95.48%)	695 (95.21%)	
Yes	102 (4.66%)	34 (4.66%)	33 (4.52%)	35 (4.79%)	
Smoking status, *n* (%)					0.57
No	2070 (94.52%)	694 (95.07%)	685 (93.84%)	691 (94.66%)	
Yes	120 (5.48%)	36 (4.93%)	45 (6.16%)	39 (5.34%)	
Alcohol consumption, *n* (%)					0.50
No	2117 (96.67%)	708 (96.99%)	701 (96.03%)	708 (96.99%)	
Yes	73 (3.33%)	22 (3.01%)	29 (3.97%)	22 (3.01%)	
Homocysteine, μmol/L	14.36 ± 8.63	8.29 ± 1.53	12.36 ± 1.28	22.44 ± 10.66	<0.001
PTH, pmol/L	13.49 ± 8.74	7.95 ± 2.02	11.45 ± 2.74	21.07 ± 11.21	<0.001
Albumin, g/L	39.91 ± 4.05	39.84 ± 3.86	40.10 ± 3.86	39.78 ± 4.41	0.26
Creatinine, μmol/L	64.77 ± 30.68	55.58 ± 13.41	62.96 ± 20.01	75.78 ± 45.12	<0.001
Uric acid, μmol/L	281.69 ± 90.69	252.07 ± 74.49	280.64 ± 84.03	312.37 ± 101.31	<0.001
Calcium, mmol/L	2.21 ± 0.13	2.20 ± 0.13	2.21 ± 0.13	2.21 ± 0.13	0.34
Phosphorus, mmol/L	1.06 ± 0.21	1.08 ± 0.18	1.06 ± 0.20	1.05 ± 0.23	0.05
Magnesium, mmol/L	0.90 ± 0.10	0.90 ± 0.09	0.90 ± 0.10	0.90 ± 0.11	0.28
ASA, *n* (%)					0.53
0	195 (8.90%)	71 (9.73%)	59 (8.08%)	65 (8.90%)	
1	1487 (67.90%)	485 (66.44%)	513 (70.27%)	489 (66.99%)	
≥ 2	508 (23.20%)	174 (23.84%)	158 (21.64%)	176 (24.11%)	
Admission season, *n* (%)					0.50
Spring	558 (25.48%)	201 (27.53%)	169 (23.15%)	188 (25.75%)	
Summer	571 (26.07%)	185 (25.34%)	196 (26.85%)	190 (26.03%)	
Autumn	550 (25.11%)	187 (25.62%)	181 (24.79%)	182 (24.93%)	
Winter	511 (23.33%)	157 (21.51%)	184 (25.21%)	170 (23.29%)	

Continuous data are presented as mean ± standard deviation (SD), and categorical variables as counts with corresponding percentages.

Group comparisons across lnHcy tertiles were assessed using one-way ANOVA or chi-square tests, as appropriate.

Abbreviations: BMI, body mass index; PTH, parathyroid hormone; ASA, American Society of Anesthesiologists classification.

### Univariate correlates of serum PTH levels

As summarized in Table 2, the univariable regression results revealed that serum magnesium and creatinine exhibited significant correlations with parathyroid hormone (PTH) concentrations. Specifically, magnesium demonstrated an inverse association with PTH (β = –6.52; 95% CI: –10.14 to –2.91; P < 0.001), whereas creatinine showed a direct positive relationship (β = 0.06; 95% CI: 0.05 to 0.07; P < 0.001). No meaningful associations were identified for age, sex, BMI, hypertension, diabetes, albumin, smoking, alcohol intake, ASA class, or season of admission (all P > 0.05). These results collectively suggest that alterations in renal performance and mineral homeostasis—particularly magnesium balance—may substantially influence PTH secretion dynamics in individuals with osteoporotic fractures.

**Table 2:** Univariate analyses of factors associated with serum parathyroid hormone (PTH).

Variable	Statistics	*β* (95% *CI*) *P*-value
Age, years	68.97 ± 10.97	0.00 (−0.03, 0.03) 0.96
Sex, *n* (%)		
Female	1647 (75.21%)	Reference
Male	543 (24.79%)	0.04 (−0.80, 0.89) 0.92
BMI, kg/m²	23.26 ± 3.28	0.02 (−0.09, 0.14) 0.68
Hypertension, *n* (%)		
No	1889 (86.26%)	Reference
Yes	301 (13.74%)	0.66 (−0.40, 1.72) 0.22
Diabetes, *n* (%)		
No	2088 (95.34%)	Reference
Yes	102 (4.66%)	−0.28 (−2.02, 1.46) 0.75
Magnesium, mmol/L	0.90 ± 0.10	−6.52 (−10.14, −2.91) <0.001
Albumin, g/L	39.91 ± 4.05	0.01 (−0.08, 0.10) 0.87
Creatinine, μmol/L	64.77 ± 30.68	0.06 (0.05, 0.07) <0.001
Smoking status, *n* (%)		
No	2070 (94.52%)	Reference
Yes	120 (5.48%)	0.17 (−1.44, 1.78) 0.84
Alcohol consumption, *n* (%)		
No	2117 (96.67%)	Reference
Yes	73 (3.33%)	0.67 (−1.37, 2.71) 0.52
ASA, *n* (%)		
0	195 (8.90%)	Reference
1	1487 (67.90%)	0.05 (−1.25, 1.36) 0.94
≥ 2	508 (23.20%)	0.43 (−1.01, 1.88) 0.56
Admission season, *n* (%)		
Spring	558 (25.48%)	Reference
Summer	571 (26.07%)	0.04 (−0.98, 1.06) 0.94
Autumn	550 (25.11%)	−0.12 (−1.15, 0.91) 0.82
Winter	511 (23.33%)	0.12 (−0.93, 1.17) 0.83

Data are presented as β coefficients (95% confidence intervals) derived from univariate linear regression models with PTH as the dependent variable.

Abbreviations: BMI, body mass index; ASA, American Society of Anesthesiologists.

### Multivariable association between homocysteine and parathyroid hormone

As summarized in Table 3, multivariable regression analyses consistently demonstrated that higher serum homocysteine concentrations were independently associated with elevated parathyroid hormone (PTH) levels. In the unadjusted model (Model 1), ln-transformed homocysteine (lnHcy) showed a robust positive correlation with PTH (β = 15.95; 95% CI: 15.51–16.40; P < 0.001). This relationship remained virtually unchanged after accounting for age, sex, and BMI in Model 2 (β = 15.95; 95% CI: 15.51–16.40; P < 0.001). Further adjustment for a broader set of clinical and biochemical variables—including hypertension, diabetes, albumin, creatinine, magnesium, smoking, alcohol use, ASA class, and admission season—did not materially alter the association (Model 3, β = 16.13; 95% CI: 15.66–16.59; P < 0.001). Taken together, these findings indicate that the association between homocysteine and PTH persisted after adjustment for multiple confounders. The underlying mechanisms remain unclear and may partly relate to metabolic or renal factors.

**Table 3:** Multivariable linear regression analyses of ln-transformed homocysteine (lnHcy) and serum parathyroid hormone (PTH) levels.

Exposure	Model 1^a^ *β* (95% *CI*) *P*-value	Model 2^b^ *β* (95% *CI*) *P*-value	Model 3^c^ *β* (95% *CI*) *P*-value
**lnHcy**	15.95 (15.51, 16.40) <0.001	15.95 (15.51, 16.40) <0.001	16.13 (15.66, 16.59) <0.001

^a^Non-adjusted model.

^b^Adjusted for age, sex, and BMI.

^c^Adjusted for age, sex, BMI, hypertension, diabetes, albumin, creatinine, magnesium, smoking status, alcohol consumption, ASA, and admission season.

Abbreviations: CI, confidence interval; BMI, body mass index; ASA, American Society of Anesthesiologists.

### Spline smoothing plot and threshold analysis

**Fig 2:**
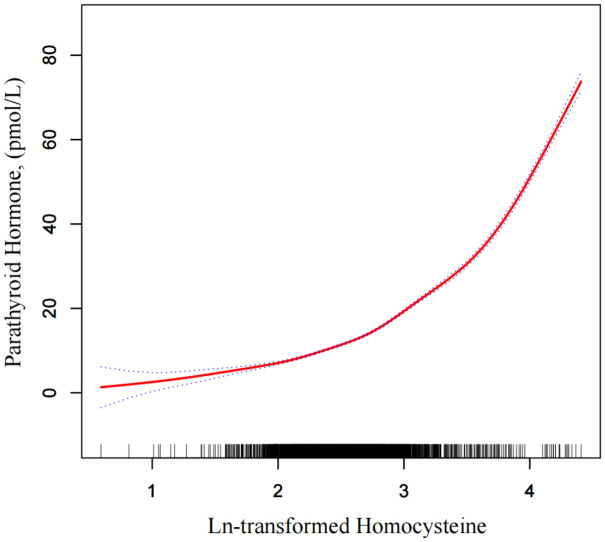
Smoothed curve of the association between lnHcy and serum PTH. Each short black tick on the x-axis represents one participant. The solid red line represents the fitted curve derived from a generalized additive model (GAM), and the blue shaded area indicates the 95% confidence interval. The model was adjusted for age, sex, BMI, hypertension, diabetes, albumin, creatinine, magnesium, smoking status, alcohol consumption, ASA classification, and admission season. Abbreviations: BMI, body mass index; ASA, American Society of Anesthesiologists.

Fig 2 and Table 4 illustrate a nonlinear association between lnHcy and PTH, indicating a clear threshold effect. Based on the fully adjusted GEE model, a smoothing spline fitted by the GAM revealed an inflection point at K = 2.91, corresponding to approximately 18 μmol/L of plasma homocysteine. Below this threshold, each 1-unit increase in lnHcy was associated with a modest rise in PTH (β = 9.27, 95% CI: 8.75–9.78, P < 0.001), whereas above the threshold, the effect became markedly stronger (β = 31.38, 95% CI: 30.50–32.26, P < 0.001).

**Table 4:** Threshold-effect analysis of the association between ln-transformed homocysteine (lnHcy) and serum parathyroid hormone (PTH) levels based on the two-piecewise linear regression model.

	Model 3^a^
	**PTH, *β* (95% CI), *P* value**
Model A^b^	
One-line slope	16.13 (15.66, 16.59) <0.001
Model B^c^	
Turning point (K)	2.91
<K segment slope	9.27 (8.75, 9.78) <0.001
>K segment slope	31.38 (30.50, 32.26) <0.001
Difference (Slope₂ – Slope₁)	22.12 (20.95, 23.28) <0.001
Log-likelihood ratio test (LRT^d^)	<0.001

^a^Adjusted for age, sex, BMI, hypertension, diabetes, albumin, creatinine, magnesium, smoking status, alcohol consumption, ASA, and admission season.

^b^Linear analysis, *P* value < 0.05 indicates a linear relationship.

^c^Nonlinear analysis.

^d^*P* value < 0.05 means Model B is significantly different from Model A, which indicates a nonlinear relationship.

Abbreviations: CI, confidence interval; BMI, body mass index; ASA, American Society of Anesthesiologists.

The difference in slopes between the two segments was statistically significant (P < 0.001), and the log-likelihood ratio test confirmed that the two-piecewise linear model provided a superior fit to the data. Overall, these findings demonstrate a threshold-dependent acceleration in the Hcy–PTH relationship, suggesting that elevated homocysteine above ~18 μmol/L may be associated with a steeper increase in PTH.

### Subgroup analysis

Subgroup assessments (Table 5) confirmed that the positive link between lnHcy and serum PTH was broadly maintained across diverse clinical categories. The association persisted among both ≤70 and >70 year age groups (P < 0.001 for each) and showed no material modification by sex, BMI, diabetes, albumin level, or lifestyle habits such as smoking and drinking (all P for interaction > 0.05). These results suggest that the observed Hcy–PTH relationship is largely homogeneous across demographic and metabolic subpopulations.

**Table 5:** Subgroup analyses of the association between ln-transformed homocysteine (lnHcy) and serum parathyroid hormone (PTH) levels according to clinical characteristics.

Subgroup	*N*	PTH, *β* (95% *CI*)	*P*-value	*P* for interaction
Age, years				0.07
≤ 70	1209	16.30 (15.69, 16.91)	<0.001	
> 70	981	15.49 (14.85, 16.13)	<0.001	
Sex				0.47
Female	1647	15.86 (15.34, 16.38)	<0.001	
Male	543	16.24 (15.42, 17.06)	<0.001	
BMI, kg/m2				0.12
< 25	1598	15.73 (15.20, 16.26)	<0.001	
≥ 25	592	16.51 (15.72, 17.31)	<0.001	
Hypertension				**0.02**
No	1889	15.74 (15.25, 16.22)	<0.001	
Yes	301	17.22 (16.14, 18.31)	<0.001	
Diabetes				0.59
No	2088	15.93 (15.47, 16.38)	<0.001	
Yes	102	16.48 (14.49, 18.48)	<0.001	
Albumin, g/L				0.61
< 40	1073	16.07 (15.46, 16.68)	<0.001	
≥ 40	1117	15.84 (15.19, 16.48)	<0.001	
Creatinine, μmol/L				**<0.001**
< 80	1864	15.70 (15.22, 16.18)	<0.001	
≥ 80	326	17.91 (16.52, 19.30)	<0.001	
Magnesium, mmol/L				**<0.001**
< 0.9	1069	16.68 (16.03, 17.32)	<0.001	
≥ 0.9	1121	15.15 (14.54, 15.76)	<0.001	
Smoking status				0.49
No	2070	15.92 (15.46, 16.38)	<0.001	
Yes	120	16.61 (15.04, 18.19)	<0.001	
Alcohol consumption				0.19
No	2117	15.90 (15.44, 16.35)	<0.001	
Yes	73	17.54 (15.98, 19.10)	<0.001	
ASA				**0.04**
0	195	14.33 (13.20, 15.46)	<0.001	
1	1487	16.28 (15.72, 16.84)	<0.001	
≥ 2	508	15.66 (14.77, 16.56)	<0.001	
Admission season				**<0.001**
Spring	558	14.74 (13.98, 15.50)	<0.001	
Summer	571	17.47 (16.58, 18.37)	<0.001	
Autumn	550	16.57 (15.59, 17.55)	<0.001	
Winter	511	15.16 (14.29, 16.04)	<0.001	

Adjusted for age, sex, BMI, hypertension, diabetes, albumin, creatinine, magnesium, smoking status, alcohol consumption, ASA, and admission season, except for the stratification variable itself.

P for interaction was derived from the multiplicative interaction term between lnHcy and the subgroup variable in multivariable linear regression models.

Abbreviations: CI, confidence interval; BMI, body mass index; ASA, American Society of Anesthesiologists.

However, several factors significantly modified the association. The relationship was stronger among patients with hypertension (P for interaction = 0.02), elevated creatinine levels (P for interaction < 0.001), and lower magnesium concentrations (P for interaction < 0.001). In hypertensive individuals, each one-unit increase in lnHcy corresponded to a greater elevation in PTH, suggesting that this association may be more pronounced in the context of vascular stress. Likewise, participants with higher creatinine levels exhibited a markedly stronger association, possibly reflecting impaired renal clearance of both homocysteine and PTH. In contrast, the association was attenuated in individuals with higher magnesium concentrations, consistent with magnesium’s regulatory role in calcium–PTH homeostasis.

Significant interactions were also observed across ASA classifications (P for interaction = 0.04) and admission seasons (P for interaction < 0.001), implying that systemic stress and seasonal metabolic variation may further influence the Hcy–PTH relationship.

## Discussion

### Key findings

In a large cohort of 2,190 osteoporotic fracture patients, plasma homocysteine showed a robust, independent, and nonlinear positive association with serum parathyroid hormone (PTH). PTH rose modestly with increasing homocysteine until an inflection at lnHcy ≈ 2.91 (~18 μmol/L), beyond which the slope steepened. Subgroup analyses indicated stronger homocysteine–PTH coupling in patients with hypertension, renal impairment, or low magnesium, suggesting that vascular, renal, and mineral homeostatic conditions may jointly modulate this relationship.

### Interpretation and potential mechanisms

Three interconnected mechanisms may explain the observed relationship. First, homocysteine promotes oxidative stress and endothelial dysfunction, reducing nitric oxide bioavailability and impairing microvascular integrity [19]. In the parathyroid gland, diminished perfusion or redox imbalance may remove inhibitory cues on PTH secretion, potentially contributing to higher PTH secretion [20]. Second, renal physiology provides another link. Homocysteine clearance depends on kidney function, while the kidney also regulates PTH degradation and vitamin D activation [21]. Accumulated homocysteine could therefore reflect or exacerbate renal disturbances, amplifying PTH secretion in a pattern resembling early CKD–mineral bone disorder [22]. Third, homocysteine directly affects skeletal remodeling by weakening collagen cross-linking and stimulating osteoclastogenesis [23]. These alterations can create a subtle decline in serum calcium, triggering a secondary increase in PTH [24]. Finally, magnesium plays a modulatory role. Mild hypomagnesemia enhances PTH secretion, while adequate magnesium stabilizes calcium–PTH balance [25]. The stronger association observed in low-magnesium individuals implies that homocysteine’s effects are accentuated when mineral homeostasis is compromised [26–27].

Collectively, these results support a vascular–renal–mineral crosstalk model, wherein homocysteine may be associated with higher PTH concentrations under concurrent oxidative, renal, and mineral stresses.

### Comparison with previous studies

Previous epidemiological studies consistently link elevated homocysteine with low bone mineral density and increased fracture risk, supported by mechanistic data showing impaired bone matrix integrity and enhanced resorption [28–29]. Some evidence suggests that PTH may mediate these effects. For example, associations between homocysteine and bone loss are attenuated after adjusting for PTH, and correlations between homocysteine and bone turnover are most evident in individuals with renal dysfunction [30]. Our findings expand upon prior research by identifying a nonlinear threshold in the homocysteine–PTH relationship and quantifying effect modification by hypertension, creatinine, and magnesium, confirming these associations in a well-characterized osteoporotic fracture cohort. While Mendelian randomization studies have not found a consistent causal link between genetically elevated homocysteine and bone density in the general population, our data suggest that such effects may emerge only in vulnerable metabolic contexts [31–32]. The stronger association in hypertensive patients supports the concept of a bidirectional relationship: homocysteine-induced vascular dysfunction elevates PTH, while chronic PTH excess contributes to arterial stiffness and hypertension, forming a self-reinforcing cycle of vascular dysfunction and skeletal fragility [33]. These results are consistent with the hypothesis that osteoporosis and vascular disease may share common inflammatory and endocrine pathways.

### Clinical significance and implications

Identifying a threshold near ~18 μmol/L may provide additional observational insight. Mild hyperhomocysteinemia is frequent among older adults due to nutritional deficiency or early renal decline [34]. Beyond this level, PTH appeared to rise more sharply, suggesting a subgroup that may warrant further investigation for secondary hyperparathyroidism, even with near-normal vitamin D and calcium levels [35]. Whether homocysteine assessment can aid metabolic risk stratification in osteoporotic patients—especially those with hypertension or impaired renal function—remains to be established. Interventional implications should be interpreted cautiously and require prospective validation, including whether lowering homocysteine, ensuring adequate magnesium, and optimizing vascular and renal health may influence PTH-related bone metabolism [36]. This integrative perspective aligns with the emerging view that bone fragility reflects not only skeletal deficits but also systemic metabolic stress [37].

### Limitations

This study has several limitations. The cross-sectional design prevents causal inference, and residual confounding due to unmeasured circulating vitamin D and B-vitamin levels cannot be excluded. We included season of hospital admission as a surrogate to partially account for seasonal variation in vitamin D, but acknowledge that this is an imperfect proxy. Our cohort included hospitalized Chinese patients with osteoporotic fractures, limiting generalizability; the identified threshold (~18 μmol/L) may differ across populations. Furthermore, we focused on intact PTH and did not assess related hormones such as FGF23 or klotho, which may also interact with homocysteine. Subgroup interactions, while biologically plausible, warrant cautious interpretation due to multiple testing and limited statistical power in smaller strata. Future studies should incorporate direct measurements of vitamin D and B-vitamins to more accurately evaluate their potential confounding effects on the Hcy–PTH relationship.

### Future directions

Prospective studies are needed to test whether elevated homocysteine predicts longitudinal increases in PTH or bone turnover and whether interventions that lower homocysteine reduce these effects. Mechanistic research should clarify how homocysteine influences parathyroid cell signaling, calcium-sensing receptor activity, and microvascular perfusion. Trials targeting patients with hypertension, CKD, or magnesium deficiency may be particularly informative, helping to determine whether integrated nutritional and vascular interventions can enhance skeletal outcomes beyond conventional anti-osteoporotic therapy [38–40].

## Conclusions

This study demonstrates a nonlinear, threshold-dependent association between homocysteine and PTH in osteoporotic fracture patients, intensified by vascular and renal stressors and low magnesium. Elevated homocysteine may represent a metabolic correlate of higher PTH concentrations in older patients with osteoporosis, although its clinical utility requires further validation.

## Supporting information

S1 File De-identified minimal dataset underlying the findings of this study. (XLSX)
